# Essential requirements for establishing and operating data trusts: practical guidance co-developed by representatives from fifteen canadian organizations and initiatives

**DOI:** 10.23889/ijpds.v5i1.1353

**Published:** 2020-08-24

**Authors:** P. Alison Paprica, Eric Sutherland, Andrea Smith, Michael Brudno, Rosario G. Cartagena, Monique Crichlow, Brian K. Courtney, Chris Loken, Kimberlyn M. McGrail, Alex Ryan, Michael J. Schull, Adrian Thorogood, Carl Virtanen, Kathleen Yang

**Affiliations:** 1 University of Toronto, Institute of Health Policy, Management and Evaluation, 155 College Street, Toronto, ON, M5T 3M6, Canada; 2 Vector Institute, Suite 710, 661 University Ave, Toronto, ON, M5G 1M1, Canada; 3 Health Data Research Network Canada, 01-2206 East Mall, Vancouver BC, V6T 1Z3, Canada; 4 ICES, G1 06, 2075 Bayview Avenue, Toronto, ON, M4N 3M5, Canada; 5 Canadian Institute for Health Information, Suite 600, 495 Richmond Road, Ottawa, ON, K2A 4H6, Canada; 6 HPC4Health, 686 Bay St. Toronto, ON, M5G 0A4, Canada; 7 University Health Network, 190 Elizabeth St., Toronto, ON, M5G 2C4, Canada; 8 Hospital for Sick Children, 555 University Ave, Toronto, ON, M5G 1X8, Canada; 9 University of Toronto, Department of Computer Science, 214 College St, Toronto, ON, M5T 3A1, Canada; 10 Compute Ontario, Suite 1140, 661 University Avenue, Toronto, ON, M5G 1M1, Canada; 11 Sunnybrook Research Institute, 2075 Bayview Avenue, Toronto, ON, M4N 3M5, Canada; 12 Population Data BC, University of British Columbia, 201-2206 East Mall, Vancouver, BC, V6T 1Z3, Canada; 13 UBC Centre for Health Services and Policy Research, 2206 E Mall, Vancouver, BC, V6T 1Z3, Canada; 14 University of British Columbia, Faculty of Medicine, School of Population and Public Health, 2206 E Mall, Vancouver, BC, V6T 1Z3, Canada; 15 MaRS Discovery District MaRS Centre, South Tower 101 College Street, Suite 100 Toronto, ON, M5G 1L7, Canada; 16 McGill University, Centre of Genomics and Policy, Suite 5200, 740, avenue Dr. Penfield, Montreal, QC, H3A 0G1, Canada; 17 Global Alliance for Genomics and Health, MaRS Centre, West Tower, Suite 510, 661 University Avenue, Toronto, ON, M5G 0A3, Canada

**Keywords:** data infrastructure, data governance, public engagement, data trust, data protection

## Abstract

**Introduction:**

Increasingly, the label “data trust” is being applied to repeatable mechanisms or approaches to sharing data in a timely, fair, safe, and equitable way. However, there is an absence of practical guidance regarding how to establish and operate a data trust.

**Aim and approach:**

In December 2019, the Canadian Institute for Health Information and the Vector Institute for Artificial Intelligence convened a working meeting of 19 people representing 15 Canadian organizations/initiatives involved in data sharing, most of which focus on public sector health data. The objective was to identify essential requirements for the establishment and operation of data trusts in the Canadian context. Preliminary requirements were discussed during the meeting and then refined as authors contributed to this manuscript.

**Results:**

Twelve minimum specification requirements (“min specs”) for data trusts were identified. The foundational min spec is that data trusts must meet all legal requirements, including legal authority to collect, hold or share data. In addition, there was agreement that data trusts must have (i) an accountable governing body to ensure that the data trust achieves its stated purpose and is transparent, (ii) comprehensive data management including clear processes and qualified individuals responsible for the collection, storage, access, disclosure and use of data, (iii) training and accountability requirements for all data users and (iv) ongoing public and stakeholder engagement.

**Conclusions:**

Practical guidance for the establishment and operation of data trusts was articulated in the form of 12 min specs requirements. The 12 min specs are a starting point. Future work to refine and strengthen them with members of the public, companies, and additional research data stakeholders from within and outside of Canada, is recommended.

## Background

Organizations around the world are actively working on ways to increase uses of person-level data for research, evaluation, planning and innovation while ensuring that data are secure, and privacy is protected [1–6]. These activities can be understood to be part of a broader effort to ensure appropriate data governance and management at a time when there is unprecedented opportunity to transform data into beneficial knowledge and significant public concern about how data are shared, protected and used. Given the potential for both public benefit and data misuse, the sharing of person level data is the focus of much debate and interest [7–12].

The term “data trust” received heightened attention when it was identified as a key mechanism to grow artificial intelligence (AI) in the UK in the 2017 Hall-Presenti report [13]. The report emphasizes the need for terms and mechanisms to facilitate the sharing of data between organizations that hold data (data providers) and organizations seeking to use data (data users). Hall and Presenti are direct in stating that the data trusts they envision are “not legal entities or institutions, but rather a set of relationships underpinned by a repeatable framework, compliant with parties’ obligations, to share data in a fair, safe and equitable way” [13].

There are other working definitions of data trusts, some of which directly contradict the definition in the Hall-Presenti report. For example, in 2020 the Open Data Institute put forward a working definition which draws upon the concept of a legal trust with trustees and beneficiaries: “a data trust provides independent, fiduciary stewardship of data” [14]. In addition, there are many other labels applied to endeavors to responsibly share and provide access to data including: digital trusts, data co-operatives, data commons, data clubs, data institutions, data banks, data stewardships, data collaboratives, data safe havens, trustworthy digital repositories and trusted research environments [15–29].

In our experience, one of the negative effects of the multiple labels and conflicting definitions is that it can obscure commonalities behind approaches to data sharing and data access. For example, the authors of this report have, at times, used several of the labels above to describe our work, while having common goals related to data that are FAIR (findable, accessible, interoperable, reusable), and well governed and managed as per the Five Safes, the recently published TRUST principles and other frameworks [27–34].

Given recent large scale Canadian public investment in data infrastructure with an initial focus on data from publicly-funded health services, our group identified a need for practical guidance about how to establish and operate data infrastructure that supports data sharing and enables access to data while continuing to ensure data protection. Since our focus was not on exclusive definitions, we modified the Hall-Presenti report language and used the working definition “a data trust is a repeatable mechanism or approach to sharing data in a timely, fair, safe and equitable way” which neither requires nor precludes the data trust taking the form of a legal entity or independent institution. Our aim was to combine our first-hand experience of Canadian data infrastructure with a synthesis of concepts from related literature to establish a common understanding of the essential requirements for data trusts, irrespective of the form that a data trust may take.

## Aim and approach

The Data Trust Working Meeting was the first “Capability Exchange” organized under CIHI’s *Health Data and Information Capability Framework* which includes “facilitating exchange of knowledge” and “exploring harmonization” as two of its objectives. Participants were invited based on their organizations’ active work on accessible data in Canada. In total 19 people representing 15 organizations and data infrastructure initiatives participated. Most participants work at publicly funded organizations focused on health data and/or data associated with publicly funded services. Several participants were involved in more than one initiative or organization, including some commercial organizations. However, to mitigate the risk that a single company could have a disproportionate influence on discussions and the outputs of the meeting, invites were not extended to representatives from commercial organizations, though it was acknowledged that companies could have multiple roles to play in data trusts including as data providers and as leaders in technology-based approaches to monitoring, security, data governance and/or provenance.

Each participating organization/initiative was asked to provide a written summary of its activities which was circulated in advance of the meeting. We held a six-hour in-person meeting in Toronto on December 3, 2019 which comprised brief (~5 minute) presentations about each organization/initiative, followed by a series of facilitated discussions. The meeting utilized a minimum specifications requirements (“min specs”) approach to identify the essential elements and key characteristics of data trusts [35]. This entailed inviting individuals to brainstorm a list of elements and characteristics that might be essential for data trusts and then, as a group, determining which should be crossed off the list based on the fact that it could be possible to have a complete and well-functioning data trust without them. Live internet polling was used to capture individual suggestions and key points from the group discussions. Preliminary min specs requirements were presented during the meeting. The min specs (Box 1) were refined as authors contributed their suggestions to a rapid literature review (performed using a snowball search method) and worked collaboratively on iterations of this manuscript.

Box 1 **Min specs for data trust establishment and operations**

1. Legal: The data trust must fulfill all legal requirements, including the authority to collect, share and hold data
2. Governance
  - The data trust must have a stated purpose
  - The data trust must be transparent in its activities
  - The data trust must have an accountable governing body
  - Governance must be adaptive
3. Management
  - There must be well-defined policies and processes for the collection, storage, use and disclosure of data
  - Policies and processes must include data protection safeguards which are reviewed and updated regularly
  - There must be an ongoing process to identify, assess and manage risks
4. Data user requirements
  - All data users must complete training before they access data
  - All data users must agree to a data user agreement that acknowledges that data use will be monitored and includes consequences for non-compliance
5. Public and stakeholder engagement
  - There must be early and ongoing engagement with stakeholders including members of the public
  - Where there is a reasonable expectation that specific subpopulations or groups would have a particular interest in, or would be affected by, an activity of the data trust, there must be direct engagement tailored for that subpopulation/group

## Results

### Requirement 1: Legal – one (1) min spec

The foundational minimum specifications requirement (min spec “1”) is that a data trust must fulfill all legal requirements. Organizations contemplating establishing and/or being part of a data trust need to be fully aware of, and be able to comply with, relevant legislation and regulations, for example with those related to collecting, using and disclosing personal information. In Canada, private-sector organizations that collect, use or disclose personal information in the course of a commercial activity must comply with PIPEDA [36]. Similar data sharing activities for public sector data must comply with provincial legislation related to privacy, such as PHIPA in Ontario [37] and FIPPA in British Columbia [38]. For that reason, fulfilling this min spec may be more complex for cross-border data trusts, as they will need to identify multiple legal requirements and ensure that governance addresses all of them.

In addition to legislation and regulations, there are typically binding terms and conditions in data sharing agreements established between legal entities when data are shared (e.g. transferred from the organization that collected the data to a separate organization that will hold data under the data trust). Finally, there will often be project specific requirements detailed in the documentation used to obtain consent from data subjects, and in the data management plan in submissions to Research Ethics Boards (REBs or equivalents (e.g. Institutional Review Boards [IRBs]).

Because new data sharing and access arrangements have the potential to go beyond what data subjects might expect, or be supportive of, legal authority to collect, hold or share data is critical. In Canada, authority will generally come in the form of at least one, and sometimes more, of the following: (i) authority defined in legislation and/or regulation, (ii) consent on the part of the data subject, (iii) the approval of an REB/IRB [39]. We emphasize legal authority here because we believe that new and widespread interest in sharing data for public benefit could lead to organizations with good intentions sharing or providing access to data without having the legal authority to do so.

### Requirement 2: Governance – four (4) min specs

The international and Canadian research literature indicates that members of the general public are conditionally supportive of data-intensive health research provided that their concerns related to privacy, security and commercial motives are addressed [9, 33, 40–44]. It is our view that governance is the best way to ensure that data trusts meet all legal requirements AND align with social licence, using the term *governance* to refer to the “locus of accountability for decision-making” in contrast with *management* which “involves making and implementing decisions” [45].

Our group identified four min specs related to governance. Foremost, min spec 2a is that a data trust must have a stated purpose. In our view it is important that the purpose goes beyond the objective of simply sharing data and aims to achieve a specific goal. Further, in the case of data related to publicly funded services, particularly data that are used without expressed consent [46], we believe that the purpose should include the goal of achieving one or more public benefit(s). For example, a data trust might have the purpose to facilitate the use of person-level data to better understand disease and wellness and evaluate health system interventions.

Data trusts must also have principles regarding how they work towards their purpose. Both the research literature and negative news coverage indicate that transparency about data sharing, e.g. what data is being used by whom for what purpose, is particularly important for public acceptance [7–12, 34, 40–44]. Therefore, min spec 2b requires that the data trust is transparent in its activities. In our view, at a minimum, data trusts should achieve informational transparency, e.g. by having easy to find plain language information about data holdings and data users for members of the public and other stakeholders.

To ensure that the purpose and principles are more than words on paper, min spec 2c requires that the data trust establishes a governing body with defined accountabilities. We specify only that there be a body (i.e. not a single person) that is accountable to stakeholders including data providers, data users and members of the public. Min spec 2c allows that the name and type of the governing body may vary (e.g. Board of Directors, Board of Trustees, Steering Committee), and the ways that responsibilities are documented (e.g. by-laws, Terms of Reference). Min spec 2c could result in data trust governance that is consolidated under the control of one (lead) organization or through a committee of representatives from partnering organizations.

The fourth and final min spec for data trust governance, 2d, is that governance must be adaptive vs. set in stone at the time of establishment. To accomplish this, the responsibilities of the governing body will generally include monitoring for unintended consequences and taking corrective action if activities do not advance the stated purpose or align with principles of the data trust. The governing body will also have to monitor and adapt to changes in legislation and regulation. Without min spec 4d, accountability could decrease over time as data sharing practices, risks and opportunities change. The requirement for adaptive governance also increases the likelihood that the data trust will identify and act on “positive risks” such as the emergence of relevant new data sources and new technologies that improve data protection.

### Requirement 3: Management – three (3) min specs

The first data trust management min spec, 3a, “there must be well-defined processes for the collection, storage, use and disclosure of data” encompasses many other more detailed requirements. It is beyond the scope of this paper to describe them all, but fulfilling this min spec will typically involve multiple auditable policies and processes including clear rules around when, how and under what authority data assets are linked or combined. As noted earlier, the Five Safes and other frameworks can provide guidance on policies and processes for data trust management, and the roles and responsibilities of various actors in the data sharing ecosystem [1, 27–34, 47, 48]. In cases where a data trust involves more than one organization, it is not necessary that all organizations have the same policies and processes. For example, two different data-holding organizations with different de-identification processes might create a joint data trust to link their data, and follow a protocol where the linkage and de-identification is performed using the processes of the organization that contributes the majority of variables to a linked dataset.

While the exact policies and processes can vary, min spec 3b notes that, at a minimum, the policies and processes of 3a must include data protection safeguards which are reviewed and updated regularly. Data protection includes measures to protect against privacy breaches (e.g. unauthorized use of data) and security breaches (e.g. attacks impugning data sovereignty or resulting in loss of control of data). Often data protection safeguards will include validation of user requests including authenticating who the user is, the data required for the scope and reason of use, and the secure environment where use will occur. Compliance monitoring should be in place to identify and respond in cases where there is insufficient protection, unintentional mistakes or deliberate malicious activities. Because threats to privacy and security will change over time, particularly cybersecurity threats, there must be a mechanism to audit privacy and security on a regular basis.

Data trust management’s need to be agile and adaptive is not limited to data protection. For example, data trust management bodies need to be aware of, and respond to, new developments in scientific methods/capabilities (e.g. the potential for artificial intelligence and machine learning to provide new insights based on multimodal data), new data sources (e.g. wearables), and changes in public sentiment related to data uses (e.g. in response to news coverage of data breaches). Accordingly, the third management min spec, 3c, is that there is a process in place to identify, assess, track, and manage risks. There are many ways to address risk including through policies, administrative processes, data governance, technology, and physical controls. The spirit of min spec 3c is that data trusts need to reassess risks continuously and establish or adapt risk responses as threats and opportunities evolve.

### Requirement 4: Data users – two (2) min specs

Much of the literature cited above focuses on the responsibilities of data holding organizations; however, it is the data users that are at the frontline of allowable/prohibited activities. To some extent, requirements related to data users are covered by mandatory policies and processes referenced in data trust management min specs. For example, an organization might have a policy that all data users must be vetted bona fide researchers and follow the practice of clear provisioning and deprovisioning of data users’ rights and access. However, to make it clear that individual data users also have responsibilities, min spec 4a requires individual data users to complete specified training before they access data. The content, length and frequency of the training would be set by the data trust governing body and/or management team and may vary, but the intent of this min spec is to ensure that all data users understand sensitivities associated with the data that they work with, and their obligations related to data use. Therefore, at a minimum, training should educate data users about the limits on how they can use data, e.g., prohibiting attempts to re-identify, barring linkage to other datasets, forbidding the sharing of login credentials, etc. There are already high-quality online training materials related to privacy, security and ethics developed by other parties [49] so data trusts would not need to develop all training materials from scratch.

In addition to training, there must be agreements that bind data users, not just the organization(s) that create, manage, and contribute data to the data trust. This is particularly important given growing concern that the processes for de-identification are not foolproof [50, 51]. To ensure accountability at the individual data user level, min spec 4b specifies that “all data users must agree to a data user agreement which acknowledges that data use will be monitored and specifies consequences for non-compliance.” The consequences can vary and may be different depending on the sensitivity of the data. For highly sensitive information, such as health data, consequences such as those that are in included in the UK Biobank material transfer agreement may be appropriate, i.e. in response to non-compliance the data trust ‘may prohibit the Applicant Principal Investigator and other researchers from the Applicant’s Institution from accessing any further data; and/or, it may inform relevant personnel within the Applicant PI’s Institution, funders of the Applicant and/or governing or other relevant regulatory bodies’ [52].

### Requirement 5: Public and stakeholder engagement – two (2) min specs

Much has been written regarding the importance of stakeholder and public engagement in data-intensive health research, and the importance of doing it well [1, 34, 40–44, 53–57]. From our perspective, members of the public are important data stakeholders who warrant tailored engagement in the same way that data holding organizations develop and nurture relationships with other stakeholders including governments, government agencies, hospitals, electronic medical record vendors, universities and research institutes, etc. In our experience, there are multiple mechanisms for active and meaningful public and stakeholder engagement, and these may change over the life of the data trust. Given the changing data sharing landscape and heightened public concern about data use, min spec 5a is simply that “there must be early and ongoing engagement with stakeholders including members of the public”, i.e. not one-time engagement or engagement that occurs after all decisions have been made.

Further, there is no single “public” [58] or single committee that can fully present the perspective of groups that are different in significant ways, for example because of a disability, a rare disease, a social determinant of health, or some other characteristic that impacts health and well-being [59–61]. For those reasons, min spec 5b notes “Where there is a reasonable expectation that specific subpopulations or groups would have a particular interest in, or would be affected by, an activity of the data trust, there must be direct engagement tailored for that subpopulation/group.” In other words, data trusts must supplement their standard engagement and involvement activities with special focused efforts for people with a special stake and concern, in particular, those facing long-standing inequities. Public and stakeholder engagement is not one size fits all.

### Guidance for implementing the min specs

A comparison between this paper and the references we cite would indicate considerable overlap and many consistencies. Our primary contribution is a distillation of ideas and guidance from the literature synthesized with our own experiences to create a relatively short list of min specs for establishing and operating data trusts. In total, we identified 12 practical but essential requirements. We concede that the count is somewhat arbitrary in that we used our judgement to combine min specs that inherently group together and separate those that might be absent from current or planned approaches to data sharing. Notably, though there were technology-related min specs, we did not find that the technological aspects of establishing and operating data trusts present a major challenge. From the perspective of organizations that already are actively working on data sharing, many of the 12 min specs are likely already fulfilled, with some exceptions.

In the case of min spec 2b “The data trust must be transparent in its activities” we are not aware of any organization involved in public sector data sharing that is intentionally opaque. However, with the limited resources available, organizations may not always prioritize work to make information about their activities public and transparent in plain language. We suggest that most organizations could fulfill min spec 2b by adopting an approach similar to the UK Health Data Research Alliance’s requirement to ‘publish a register of active projects accessing the data under their custodianship and new data access requests received’ [62] since the published information would be also gathered as part of routine data trust operations.

Regarding min spec 4b, “All data users must agree to a data user agreement which acknowledges that data use will be monitored and includes consequences for non-compliance”, in our experience it is standard practice to have data user agreements signed by researchers and trainees, but there has not been a strong emphasis on individual consequences for non-compliance. If, as the Hall-Presenti report suggests, the goal is widespread sharing and use of data, the future will involve hundreds to thousands of new data users. Among these users, some will make unintentional mistakes and a small subset will be bad actors. In response, we will need consequences for non-compliance that are one step down from the organization level to hold individuals accountable (not just the organizations that they belong to) with consequences that are aligned with the severity and intent of their actions.

In the case of min specs 5a and 5b, we find that most organizations with data infrastructure do have some mechanisms for engaging their stakeholders including members of the public; however, it may be treated as a parallel activity vs. one that is integrated into data sharing activities. For example, health data are often collected and shared by hospitals which have patient and family advisory committees. In such cases, it would be a small but necessary step to establish new ongoing mechanisms to inform, consult or involve stakeholders and fulfill min spec 5a. Further, acknowledging that there is no single “public”, min spec 5b might, under certain circumstances, require some organizations that are sharing data to go beyond their usual group of advisors, with targeted engagement and involvement for groups and subpopulations with different needs and interests. We also recommend further engagement to ensure that the labels applied to various forms of data sharing are intuitive and resonate with members of the public.

### Beyond min specs

At the Capability Exchange in December 2019, 288 individual comments and suggestions were captured covering a range of requirements that could be applied to data trusts. More than 85 per cent of the comments were incorporated into the 12 min specs presented in this manuscript (Box 1). Most of the suggestions that were not incorporated into the min specs were related to the involvement of commercial organizations in data trusts (see Future Work). During the Capability Exchange meeting and preparation of the manuscript, there was agreement on five additional elements which did not meet our stringent requirement for min specs (i.e. we felt that it could be possible to have a complete and well-functioning data trust without them) but were seen as highly desirable where feasible:

- Dynamic consent for data subjects (in cases where data require consent for collection) [63]
- Data traceability so that data trusts can fully execute on patient consent withdrawal, bias monitoring, audits, and regulatory agency review [64, 65]
- Standard and computable data use conditions [66]
- Secure and auditable computing environments [67]
- Public engagement that goes beyond informational transparency and into activities like co-design and deep involvement of data subjects in governance [53–60, 68–70]

The fact that these and other potential requirements for data trusts are not included in our list of min specs (Box 1) does not mean that they are unimportant add-ons. It is possible that these and other requirements become the norm as threats and opportunities related to data sharing increase, and as technological approaches to data protection mature and become more widespread. Our stringent criteria regarding what constitutes a min spec is based on our first-hand experience with data infrastructure. In practice, it is necessary to find a balance between totally locked-down data and/or extensive technological control of data with ease of use and the cost to establish and maintain data infrastructure. Even for light-touch governance and management for non-sensitive data, there still needs to be funded staff to ensure the provisioning of users, security protocols, public engagement etc. Data infrastructure, especially distributed data infrastructure, may not have the look of traditional large-scale research infrastructure like wet labs, large microscopes, and other scientific equipment, but it still needs to be funded. Accordingly, we have identified the min specs that we believe are essential requirements with the hope that focusing the available funding on them will enable the most, and the most responsible, data sharing possible with the finite resources that are available.

### Future work

For the most part, the 12 min specs presented in this manuscript reflect the perspectives and experiences of Canadians who have occupations related to the policy and operations of public sector health data infrastructure. We take it as a given that the min specs list would be improved by contributions from other people with different perspectives.

Foremost, it is our view that recommendations which would affect or involve any particular group should be co-developed with representatives of that group. Accordingly, min specs 5a and 5b related to public engagement must be reviewed and refined with members of the public. This will entail the creation of new materials and engagement forums, co-created with members of the public for members of the public, rather than a simple reuse of the materials and approach used in the December 2019 Capability Exchange. Importantly, engagement with the public will not be limited to the review of min specs 5a and 5b. We will seek advice from members of the public about improvements to the full list of min specs, including the possibility that additional min specs are required.

Secondly, there are many other organizations and initiatives working to improve data sharing in Canada and in other countries. Within Canada, we plan to begin using and evaluating the 12 min specs in our own activities, while publicizing them and encouraging their use by others. We expect that this will result in some changes and clarifications to the min specs. We also note that though the 12 min specs were developed by Canadians for the Canadian context, there is nothing inherent in the min specs that would limit their application to Canada. For example, min spec 1 “The data trust must fulfill all legal requirements, including the authority to collect, share and hold data” is very similar to the first requirement of a recent report from the Open Data Institute: “There are some things that a data institution must do, for example to comply with laws and regulations” [34]. While it is beyond the scope of this paper to consider if or how the 12 min specs may need to change to reflect international legal requirements (e.g. in the General Data Protection Regulation (GDPR) [71]) or international standards (e.g., ISO/IEC 27014 [72]), we would welcome the opportunity to partner with international colleagues on such work. To start we might focus on harmonized data trust guidance that organizations in various countries could apply independently, noting that harmonized guidance could be the foundation for min specs that can be applied to international data sharing.

Thirdly, as noted in the AIM AND APPROACH section of this manuscript, commercial organizations were not invited to participate in the Capability Exchange in order to mitigate the risk that a single company could have a disproportionate influence. Collectively, we have some awareness of the advances that commercial organizations are making with products and services related to data sharing, data provenance and data security; but acknowledge that our knowledge of leading edge commercial technologies is incomplete, particularly when technologies are used in other sectors (e.g. banking). Therefore, a dedicated and tailored engagement with companies is recommended to review and refine the min specs and potentially establish one or more new requirements related to company involvement.

In closing, in the same way that min specs 2d, 3c and 5a emphasize the need for ongoing work and adaptive governance and management of data, we see our min specs list as a dynamic set of requirements that will evolve over time. This manuscript is intended to be a helpful starting point, which we look forward to refining and improving with contributions from many others.

## Conclusions

Based on our experience with data infrastructure in Canada, we identified a relatively small number (12) of min specs for establishing and operating data trusts which should be practical to implement. The mechanism of a Capability Exchange combined with min specs facilitation was effective for identifying essential requirements for data trusts. This feature paper is just a start; continued joint work with members of the public, representatives from commercial organizations and from other Canadian and international organizations involved in data infrastructure is recommended on this evolving topic.

## Acknowledgements

We thank Alexandre Le Bouthilier, co-lead of the Terry Fox Research Institute/Imagia Digital Health and Discovery Platform funded by the Canadian government, who could not attend the December meeting but contributed to this manuscript. We also thank Frank Gavin, a Canadian patient and public advisor who is Chair of the Public Advisory Council of Health Data Research Network Canada, for his review of the draft manuscript and contributions which improved its clarity.

## Ethics statement

This manuscript did not involve primary research on human subjects and was not submitted for Research Ethics Board approval.

## Abbreviations

AI	artificial intelligence
EHDEN	European Health Data & Evidence Network
FAIR	findable, accessible, interoperable, reusable
FIPPA	*Freedom of Information and Protection of Privacy Act*
GDPR	*General Data Protection Regulation*
HDR UK	Health Data Research UK
ICES	Institute for Clinical Evaluative Sciences
IEC	International Electrotechnical Commission
IJPDS	International Journal of Population Data Science
ISO	International Standards Organization
IRB	Institutional Review Boards
ML	machine learning
NHS	National Health Services
OECD	Organization for Economic Co-Operation and Development
PI	Principal Investigator
PIPEDA	*Personal Information Protection and Electronic Documents Act*
PHIPA	Personal Health Information Protection Act
REB	Research Ethics Boards
UK	United Kingdom
